# Current considerations in prostate artery embolisation

**DOI:** 10.1186/s42155-026-00689-5

**Published:** 2026-04-22

**Authors:** Peter Mark, Nicholas I. Brown, William E. L. Ormiston

**Affiliations:** 1 Department of Interventional Radiology, Sir Charles Gairdner Hospital, Nedlands, WA Australia; 2 The Wesley Hospital, Brisbane, QLD Australia; 3 The University of Queensland, Brisbane, QLD Australia

**Keywords:** Prostate artery embolisation, Benign Prostatic Hyperplasia, Liquid embolic, Microparticles, Planning CTA, Cone beam CT, Radial Access, Prostate volume, Preoperative biomarkers, Surgical adjunct

## Abstract

**Background:**

Prostate artery embolisation is an increasingly common, minimally invasive procedure performed for lower urinary tract symptoms caused by benign prostatic hyperplasia, with numerous systematic reviews and meta-analyses concluding that PAE is safe and effective. Despite mounting evidence supporting its role in treating BPH, variability and debate exist across various practical and technical factors associated with PAE.

**Main body:**

PAE requires the use of a permanent embolic, typically microparticles or a liquid embolic, to induce ischaemia within the transitional zone of the prostate and shrink hyperplastic nodules. Microparticles have a large body of evidence that has established them as safe and effective. Liquid embolics can reduce procedure time and radiation dose but require operator experience with preparation and delivery. Pre-procedural CTA and cone beam CT are useful in defining anatomy and identifying non-target vasculature with the aim to reduce procedure time and risk of non-target embolisation but are associated with increased radiation dose. PAE can be safely performed from either a femoral or radial approach. Femoral access may be associated with higher rates of local complications and radial access is generally preferred by patients, although patient anatomy should be considered when selecting the site of access. There is recent evidence supporting the use of PAE as first-line therapy for BPH or as an adjunct prior to surgical intervention.

**Conclusion:**

Prostate artery embolisation is an important treatment option within the algorithm for managing BPH, and has matured into a credible, evidence-based option alongside surgery and medications. While the efficacy and safety of PAE are well established, varied technical considerations exist regarding embolic choice, access route, imaging adjuncts and the procedure’s role within the treatment sequence. Current data supports a pragmatic, anatomy-driven approach that prioritises procedural precision, embolic control, and patient-centred decision-making over adherence to a single technique.

## Background

Prostate artery embolisation (PAE) is an increasingly popular minimally invasive procedure performed for lower urinary tract symptoms caused by benign prostatic hyperplasia (BPH). Historically, PAE was first performed in the setting of intractable haematuria but early operators noticed subsequent improvements in lower urinary tract symptoms [1]. The procedure has now evolved into a low-risk, effective and durable non-surgical treatment for obstructive BPH that can be repeated and does not preclude any other future treatment option. As the evidence supporting PAE strengthens, variability has emerged regarding technical aspects related to performing this procedure.

Benign enlargement of the prostate gland occurs naturally due to unregulated hyperplastic growth of the epithelial and fibromuscular tissues of the transition zone (TZ) from testosterone and its metabolite, dihydrotestosterone, as well as dehydroepiandrosterone, estradiol, insulin-like growth factors and inflammatory markers (such as CRP) [2, 3]. The enlarged TZ encroaches on the urethra and bladder, contributing to bladder outlet obstruction [4]. More than half of men aged between 51 and 60 years show pathological features consistent with BPH on autopsy [3], and symptomatic BPH is present in up to 80% of men older than 70 years [2, 5]. Of these, 25% experience moderate to severe lower urinary tract symptoms (LUTS) such as poor urinary flow, frequency, hesitancy initiating flow, post-void dribbling and nocturia impairing their quality of life [3].

Current BPH management is multimodal with both pharmacological and surgical treatment options used to improve urinary function and reduce LUTS in randomised controlled trials [6]. First-line management typically involves lifestyle factors and pharmacologic therapy. A variety of pharmacological agents are utilised including alpha-adrenergic antagonists, beta-3 adrenergic agonists, 5-alpha-reductase inhibitors, anticholinergics, vasopressin analogues, phosphodiesterase-5 inhibitors and phytotherapeutics [7], with combination α−1 blocker and 5-alpha-reductase inhibitors a common first-line approach [7]. However, medication alone is often insufficient for long-term management, with up to 30–50% of men discontinuing treatment due to inadequate symptom control or intolerable adverse effects. Transurethral resection of the prostate (TURP) is the standard of care, and the benchmark for which all other therapies are compared [8, 9]. For larger glands open surgery or endoscopic enucleation can be performed [10]. TURP is associated with short-term morbidity of 11.1%, including urinary retention, surgical revision, UTI and bleeding requiring transfusion [11]. Late complications include incontinence, bladder neck contracture, urethral strictures, retrograde ejaculation and erectile dysfunction [11]. Multi-morbid and elderly population are more prone to perioperative complications [12]. In light of poor results from pharmacological therapy and morbidity associated with surgical techniques, newer treatment options have emerged [1].

Prostate artery embolisation was first performed as a treatment for BPH over 25 years ago [1]. It is a minimally invasive, non-surgical procedure performed by interventional radiologists that involves delicately blocking the prostatic arteries using microparticles or liquid embolic, resulting in targeted ischaemia and progressive reduction in gland volume, which reduces compression on the prostatic urethra and bladder [13]. Numerous systematic reviews and meta-analyses have concluded that PAE is safe and effective, and provides sustained improvements in IPSS, peak urinary flow (Qmax), post-void residual (PVR) and quality of life (QOL) [5, 10, 14, 15]; however, variability in technique persists.

### Choice of embolic and technique

**Fig. 1:**
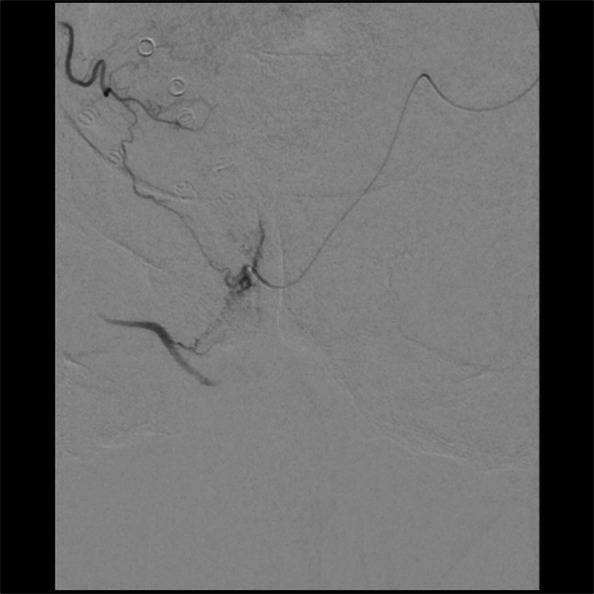
Small intraprostatic shunt with non-target internal pudendal artery collateralisation

Prostate artery embolisation seeks to achieve complete stasis of the arteries supplying hyperplastic nodules in the transitional zone, typically with microparticles (PVA or calibrated microspheres) or N-butyl cyanoacrylate-based (NBCA) glue. Early studies utilised microparticles to demonstrate safety and efficacy of PAE, and these are consequently the most widely employed embolic [16–18]. A consensus on the ideal size for microparticles has not yet been established, with sizes ranging from 100 μm up to 500 μm having been demonstrated to be efficacious. Torres et al. (2019) conducted a prospective randomised trial comparing 100–300 μm and 300–500 μm embospheres and found no difference in clinical outcomes with an increased risk of transient dysuria in the smaller microsphere group [19]. Wang et al. (2018) conducted an RCT comparing 50 μm plus 100 μm PVA particles with 100 μm PVA particles alone; MRI in the following week demonstrated higher proportions of ischaemia in the 50 μm plus 100 μm particle group [20]. A systematic review and meta-regression performed by Geevarghese et al. (2020) demonstrated that smaller particle size was associated with significantly greater reductions in IPSS at 12 months [21]. It is theorised that the smaller particles penetrate more deeply into the capillary bed to provide a more ‘distal’ and robust embolisation, although this added efficacy is weighed against a potential increased risk of more serious complications from non-target embolisation via small shunts/collateral pathways, see Fig. 1. Whilst more distal penetration of smaller particles may also increase the prevalence of post-embolic symptoms such as dysuria and urinary frequency, the incidence of clinically significant off-target embolisation remains rare [19].

The ‘PErFecTED’ technique refers to “proximal embolisation first, then embolize distally”. This method of microparticle delivery involves initial embolisation of prostate tissue from a proximal position along the prostatic artery followed by repositioning of the microcatheter more distally to complete the embolisation within the prostatic parenchyma [22]. This technique aims to achieve more complete devascularisation of the prostate by increasing the volume of embolic that can be injected [22]. Advocates of this technique argue it leads to better embolisation of the distal periurethral branches and potentially increases the treatment effect and durability of PAE [22]. Premature stasis can result from proximal embolisation and Carnavale et al. (2014) estimate an additional 30–50% of microparticles can be delivered by repositioning the catheter distally when this occurs [22, 23]. However, this technique may not be required in every case, nor essential to achieving a therapeutic effect. Its use depends on operator experience and anatomical feasibility and it may not always be possible to position the microcatheter more distally once the proximal embolisation has been done.

**Fig. 2:**
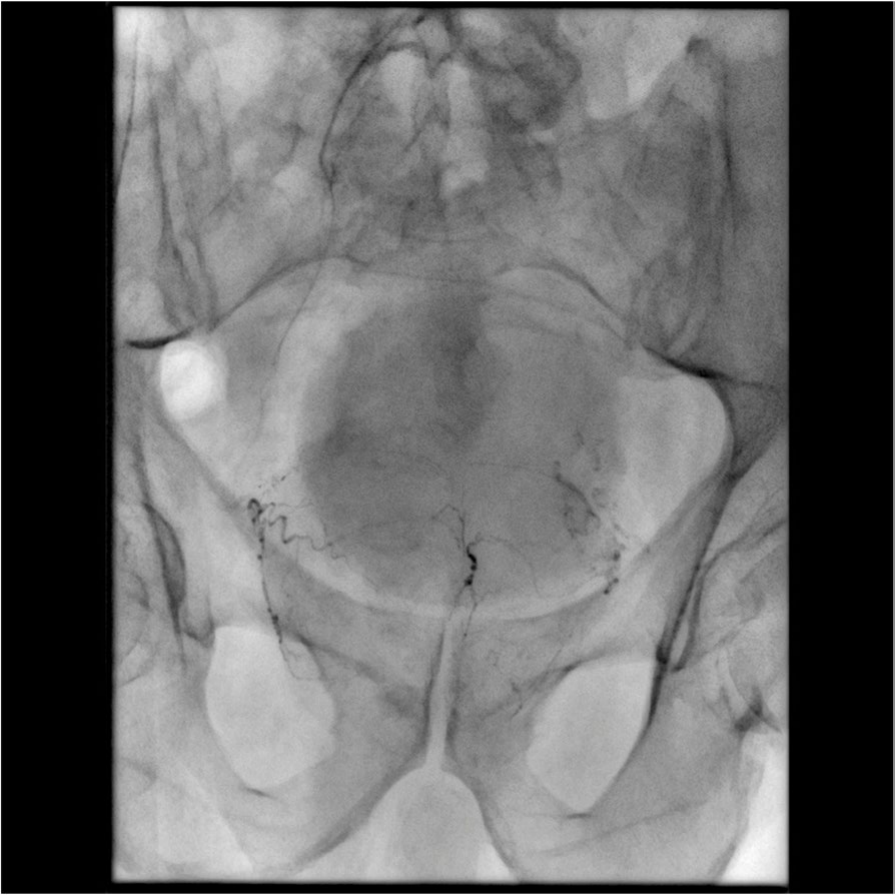
NBCA glue injection

Liquid embolics, in particular NBCA glue (histacryl (B Braun) and Glubran (GEM Italy), have emerged more recently as alternatives to particulate embolics. A dilute mixture of NBCA and iodized oil (lipiodol) of at least 1:6 is recommended to ensure distal embolisation and reduce the risk of reflux or non-target embolisation [17, 24] (Fig. 2). The potential benefits of NBCA include reduced procedure time, fluoroscopy screening time and radiation dose; however, effective use of NBCA requires significant operator experience in preparation and delivery to ensure adequate target embolisation and avoid non-target complications [17, 24]. A small case series demonstrated the safety and utility of liquid embolics using ethylene vinyl alcohol (EVOH, “Squid 12” (Balt Group) [25]. The rapid polymerisation of liquid embolics is thought not to leave sufficient time for pre-existing vascular anastomoses to open and therefore reduces the likelihood of non-target embolisation [24]. Salet et al. (2022) found no difference in complications between microspheres and NBCA in their retrospective study [17]. Potential disadvantages of liquid embolics include difficulty controlling the injection as well as a permanent blockade of the prostate artery that prevents repeat treatments in the future if symptoms recur, although it has been suggested that this could also help to improve durability of clinical improvements. It should be noted that the long-term implications of permanent prostate artery occlusion are not fully understood. Prospective comparative trials between the two embolic options are lacking and clinical outcomes to date have not been shown to be significantly affected by the choice of embolic agent.

### Use of cone beam CT and pre-procedural CT angiography

**Fig. 3:**
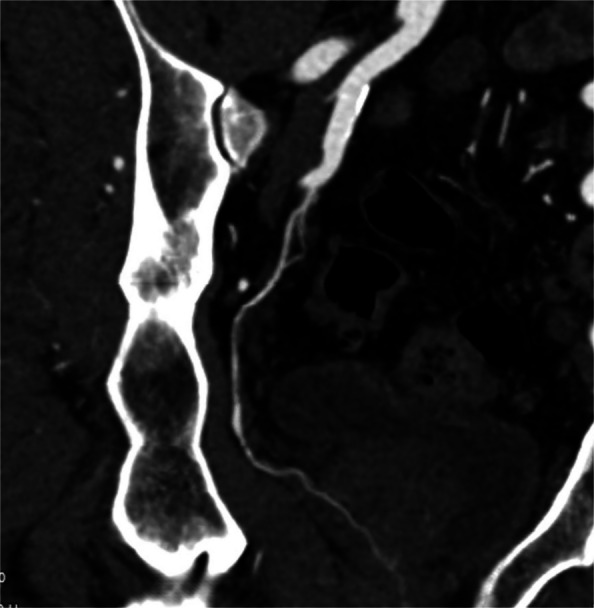
Prostate artery identification of planning CTA

**Fig. 4:**
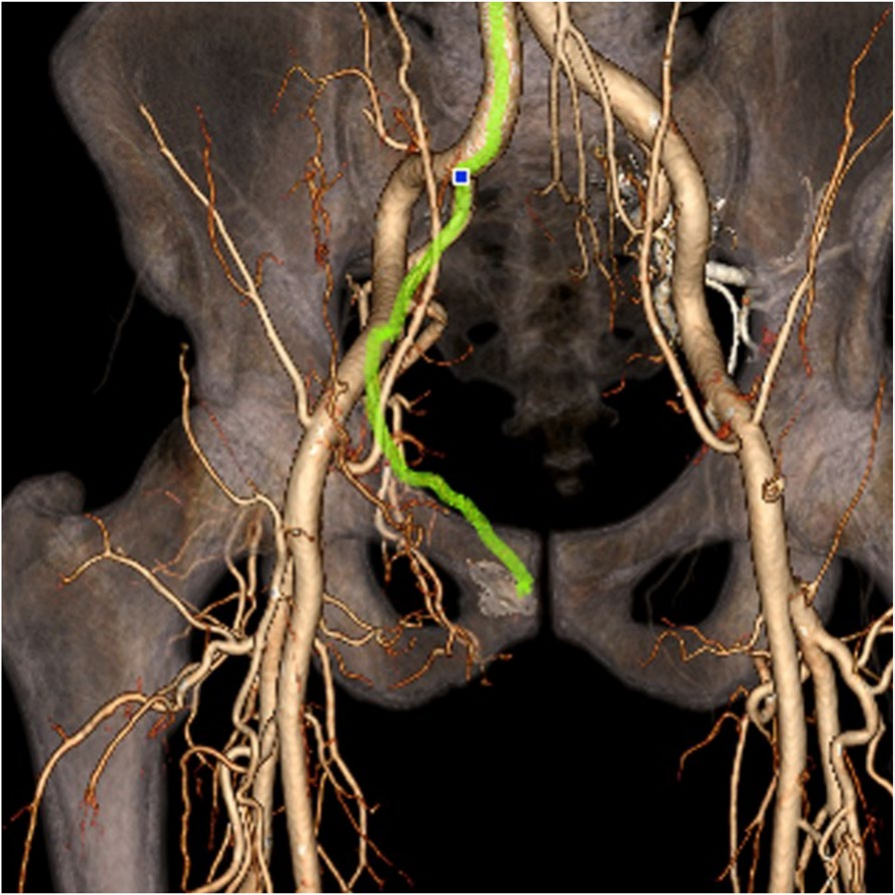
CTA reformat, planning for fusion

**Fig. 5:**
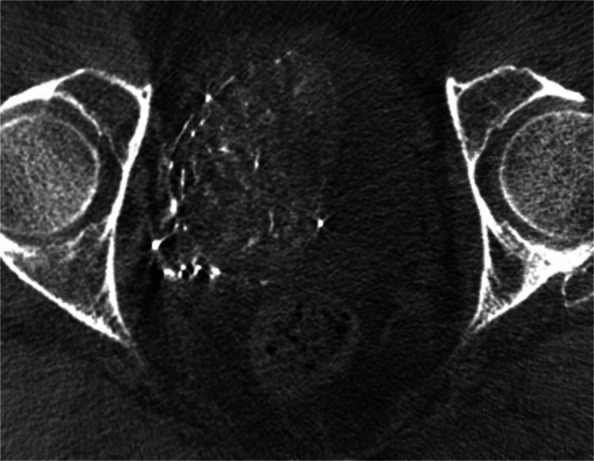
CBCT from right prostate artery injection

**Fig. 6:**
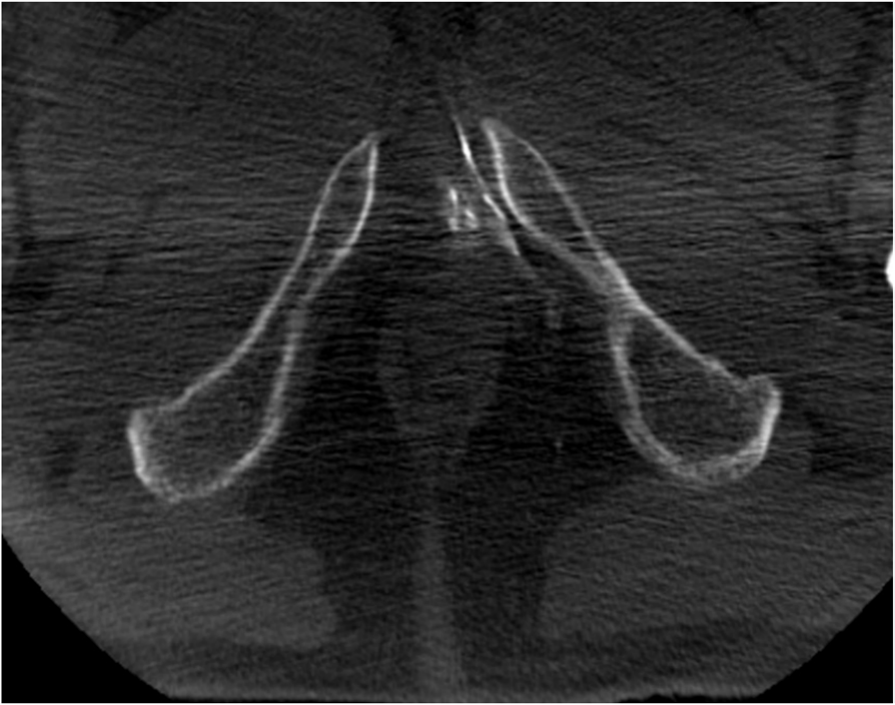
CBCT demonstrating non-target penile enhancement

One of the major technical challenges of performing PAE is the identification of and navigation through the highly variable pelvic and prostatic vascular anatomy [26, 27]. Prostate arteries are small, often tortuous and commonly stenotic vessels that have unpredictable origins and multiple anastomoses [26, 27]. Non-target embolisation is rare, transient and usually self-limiting, but can induce ischaemia in structures such as the rectum, bladder and penis [10, 15, 26]. Non-target embolisation can result from inadequate workup or misinterpretation of the blood supply to the prostate [28]. Performing a pre-procedural CTA allows for identification of the prostate arteries prior to the procedure and aids in planning (Figs. 3 and 4) [29]. Maclean et al. (2018) demonstrated that pre-procedural CTA optimised with glyceryl trinitrate (GTN) administration successfully identified the correct prostatic arterial supply in 97.3% of CTAs performed (214/220) [29]. Pre procedural CTA allows for fusion CT to be used intra-procedurally to quickly access the internal iliac arteries and for calculation of optimal tube angulation to clearly visualise the prostate artery origin on subsequent angiogram [29]. Cone beam CT can also be used to accurately identify the prostate artery at time of PAE [26] and reduce the risk non-target embolisation, see Figs. 5 and 6 [30]. Whilst not essential CBCT and pre-procedure CTA can improve the safety of PAE and reduce the overall procedure time, total screening time and radiation dose, despite the added time and radiation required to complete them.

### Access

PAE can be safely performed from either femoral or radial access [13, 31]. Femoral access remains the more common approach for interventional radiology endovascular procedures, but may be associated with higher rates of access site complications, such as haematoma and pseudoaneurysm [13, 31]. The transradial approach allows for earlier ambulation and hospital discharge compared to the transfemoral approach and is generally preferred by patients [31, 32]. Radial artery occlusion incidence post transradial access was reported in one study at 4.4%, but the risk reduces with the use of heparin, slender sheaths, and smaller catheter sizes [33]. Other recent studies found no significant difference in adverse events, radiation exposure, procedural time, or clinical outcomes with radial access compared to femoral access [13, 31].

Ultrasound, the Barbeau test or other methods for assessing the patency of collateral arterial supply to the hand are recommended prior to radial access to reduce the risk of ischaemic complications [32, 33]. A TIA or stroke is a rare but important potential complication to arise from transradial access; this may occur from dislodging atherosclerotic plaque or thrombus formation in the aortic arch or proximal subclavian artery [13, 34]. The MOSAIC study by Koretsune et al. (2025) found 40% of patients undergoing transradial hepatic intervention had asymptomatic silent brain infarcts on MRI brain post procedure. This was reduced to 2.9% by excluding patients with unfavourable arch anatomy and incorporating continuous heparinisation and catheter perfusion [35]. Richardson et al. (2024) reported an incidence of 0.2% (2 out of 821) for TIA from transradial access for PAE [13]. The risk of a cerebral event must be considered when selecting patients for transradial access with significant supra-aortic atherosclerotic disease.

In tall patients, catheter length requirements may present technical challenges with transradial access, with currently available microcatheters designed specifically for radial-to-prostate access ranging from 165 to 175 cm in length. Unfavourable aortic arch anatomy may make radial access more challenging, with significant vessel tortuosity reducing torqueability and trackability of catheters. Conversely, transradial access may be preferred when navigating tortuous common iliac arteries or acute aortic bifurcations [36].

Angiography machine and room setup is another consideration when planning access and considering a transradial approach. Distal radial puncture at the anatomical snuff-box is an alternative radial artery approach that can help to overcome technical limitations inherent in the orientation of some angiography units, especially acquisition of cone-beam CTs over the pelvis. Bi-plane angiography units are not typically necessary for PAE procedures, but can be utilised when available.

### PAE as first line therapy

Medical therapy is standard first-line treatment of obstructive BPH [6, 8], especially combined medical therapy with Dutasteride and Tamsulosin. Dutasteride is a 5α-reductase inhibitor reducing dihydrotestorone production in the body [7]. Tamsulosin is a selective α1 receptor antagonist and acts to cause smooth muscle relaxation in the bladder, prostate, and urethra [7]. Combination therapy in the CombAT trial demonstrated a 6.2 point reduction in IPSS score [7]. However, medical therapies are associated with a range of adverse effects that limit their use. Common AEs for dutasteride relate to gynaecomastia and sexual dysfunction, including impotence, altered libido, and ejaculation disorders [37]. Tamsulosin use is associated with headaches, dizziness, orthostatic hypotension, and syncope [6, 37], which can then lead to secondary adverse outcomes such as falls [37].

PAE is typically considered a second-line treatment option for patients who have received inadequate benefit from, or not tolerated, medical therapy and who are not surgical candidates or have declined surgery [38]. Two recent randomised control trials provide evidence that PAE could be considered a safe and effective early treatment for BPH, and a potential alternative to first-line medical therapy [38, 39]. The P-EASY ADVANCE trial compared PAE to combined medical therapy as first-line treatment in treatment-naïve patients. PAE was superior to combination therapy, with greater reductions in IPSS (−13 vs −6.5), improved QOL (−2.9 vs −1.4), increased Qmax and greater reduction in prostate size [38]. 87% of the medical therapy group subsequently proceeded to PAE due to inadequate treatment response from medication, and achieved a further reduction in IPSS of 4.2 points [38]. The PARTEM trial also compared PAE to combined medical therapy in patients who had previously failed medical treatment. This study also demonstrated statistically significant superiority of PAE compared to medical therapy with a reduction in IPSS of −10 for PAE vs −5.7 for combined medical therapy [39]. Both of these studies highlight the benefits of considering PAE as an alternative first-line treatment for BPH; however long-term data is lacking, and further randomised control trials are required to evaluate whether early intervention with PAE can prevent progression of BPH and associated bladder dysfunction more effectively than medications.

PAE involves exposure to ionising radiation, and doses can be influenced by many factors, including patient size, vascular anatomy, the presence of atherosclerotic disease, procedure time and interventional radiologist experience. Most PAE procedures produce skin doses of between 1.0 and 2.0 Gy across a 360° arc, with median effective doses of between 12.3 mSv and 17.8 mSv, which is equivalent to or less than most multiphase diagnostic CT scans of the abdomen and pelvis [40]. A retrospective analysis of PAE by Ayyargi et al. (2024) calculated an average DAP of 161.8 Gy.cm^2^, with a probability of cancer-related death from the intervention of 0.07–0.09%. They found no radiation-related adverse events [40]. A study by Zumstein et al. (2021) calculated a mean dose area product (DAP) of 181.6 Gy∙cm^2^ for PAE procedures, with a probability of cancer death from the intervention of 0.117%. Given the advanced age of most PAE patients, the impact of stochastic radiation effects on this population is likely to be lower than this with doubtful impact on life expectancy [40]. While the risks from radiation are very low, they are not negligible and safe radiation practices should be employed to keep doses as low as possible [40, 41].

### PAE as a surgical adjunct

Large prostate volumes present technical challenges for many surgical techniques, with higher morbidity and resection times associated with operations performed on prostate glands above 100 cc [42]. In prostates larger than 80 cc, Holmium laser enucleation of the prostate (HOLEP) and retropubic prostatectomy are surgical alternatives to TURP [9, 43]. Several case series have demonstrated safety in operating on prostate glands after PAE [44, 45]. PAE performed prior to surgical intervention, especially in very large and vascular prostates, can reduce prostate size to improve operative access and reduce bleeding risk. Lee et al. conducted an RCT evaluating the impact on intraoperative blood loss by performing PAE immediately before TURP for prostate glands larger than 80 cc. Patients were randomised to undergo PAE the morning of their TURP or TURP alone, with reduced intraoperative blood loss resulting in the PAE group [43]. Although pre-operative embolisation in this study did not impact resection efficiency or complication rates [43], a longer time interval (months) between PAE and TURP would be required to allow for pre-operative downsizing of the prostate volume to further optimise operative conditions. Although further studies are ongoing, PAE prior to surgical or focal treatment should be considered a valid option in challenging large prostate glands. PAE can also be considered in the setting of prostate cancer as neoadjuvant therapy to downsize the prostate volume prior to planned prostatectomy or radiotherapy and also to treat LUTS for patients undergoing active surveillance [46–48].

### Prostate size threshold

Prostate volume and severity of LUTS symptoms are often not directly correlated, reflecting the multifaceted causes of LUTS and variable bladder function associated with BPH. The CIRSE Standards of Practice on Prostate Artery Embolisation recommends the procedure for prostates greater than 30 cc [23]. Many papers define a minimum prostate volume of 40–50 cc as a reasonable threshold for PAE. Larger prostates are more likely to have a greater reduction in volume from embolization [23], although there is mixed evidence regarding whether this translates to improved symptomatic reduction after PAE [23, 49, 50]. A prospective 2018 study of 86 patients who underwent PAE using linear regression analysis found that initial prostate size was associated with greater clinical improvement [50]. A more recent 2024 study retrospectively assessed the impact of prostate size on IPSS outcomes in 65 patients and found no significant difference in mean change in reduction in IPSS for prostate volumes less than 51 cc vs greater than 51 cc (10.2 vs 11) [49]. Patients with prostates < 30–40 cc should be informed prior to treatment about potential reduced efficacy of PAE and likely earlier recurrence, but should not be precluded from treatment.

### Preoperative biomarkers

Identification of pre-procedural biomarkers to predict response to PAE is an evolving and important research area, particularly in the context of 10–30% treatment failure or early recurrence. Baseline parameters such as prostate volume, especially transition zone volume and PSA elevation that correlate with a more adenomatous-dominant gland, may predict greater post-PAE therapeutic effect. For example, in a comprehensive review of predictors, higher baseline prostate volume and early PSA rise (24 h post-embolisation) were associated with better symptom improvement and prostate volume reduction [51]. In addition, functional imaging biomarkers such as dynamic-contrast enhanced MRI or CT perfusion have shown promise in identifying perfusion heterogeneity and vascular bed density within the gland, which may influence infarction success following embolisation. Identification of enlarged median lobes and intravesical prostatic protrusion is also relevant, as these can be associated with sub-optimal outcomes after PAE [52]. In one study, pre-interventional CT perfusion parameters significantly correlated with PSA change at 24 h post-PAE and subsequent volume reduction [53]. Although systemic inflammatory markers (for example CRP or neutrophil–lymphocyte ratio) are hypothesised to reflect underlying prostatic inflammation that could impact embolisation outcomes, the data remain preliminary [54]. In clinical practice, a pragmatic approach that considers PSA, gland volume (including transition zone index) and imaging biomarkers in concert to aid patient selection, procedural planning and counselling, rather than as definitive predictors of success.

## Conclusion

Prostate artery embolisation is a valid and important treatment option within the algorithm for managing BPH and has matured into a credible, evidence-based option alongside surgery and medications. While the efficacy and safety of PAE are well established, technical controversies/deliberations persist regarding embolic choice, access route, imaging adjuncts and the timing of embolisation within the treatment sequence. Current data support a pragmatic, anatomy-driven approach that prioritises procedural precision, embolic control, and patient-centred decision-making over adherence to a single technique. The emergence of new embolics such as NBCA, the expanding use of transradial access, and evidence for PAE as a first-line or preoperative adjunct highlight the ongoing evolution of the field and the need for further studies. Future research should focus on improving technical aspects, patient selection, predictors of response through imaging and biomarker profiling, and evaluating long-term outcomes against contemporary surgical and medical results. As expertise and evidence continue to grow, PAE is poised to become an integral component of comprehensive, minimally invasive prostate care.

## Data Availability

Available.
